# Serum hyaluronic acid and procollagen III, N-terminal propeptide levels are highly associated with disease severity and predict the progression of COVID-19

**DOI:** 10.3389/fcimb.2023.1249038

**Published:** 2023-10-04

**Authors:** Ti Yang, Le Le Liu, Xin Han Wu, Jian Guo Xue, Chun Yan He

**Affiliations:** Department of Clinical Laboratory, Kunshan Hospital of Chinese Medicine, Affiliated Hospital of Yangzhou University, Kunshan, China

**Keywords:** COVID-19, HA, PIIIN-P, severity, marker

## Abstract

**Background:**

The coronavirus disease 2019 (COVID-19) pandemic is a rapidly evolving global emergency and continuously poses a serious threat to public health, highlighting the urgent need of identifying biomarkers for disease severity and progression. In order to early identify severe and critical patients, we retrospectively analyze the clinical characteristics and risk indicators of severe disease in patients with corona virus disease 2019 (COVID-19).

**Methods:**

A total of 420 confirmed COVID-19 patients were included in the study. According to the “Diagnosis and Treatment of novel coronavirus Pneumonia (10th Edition)”, the cases were divided into mild group (n = 243) and severe group (n =177). Laboratory parameters were analyzed in combination with clinical data.

**Results:**

Male patients over 46 years who have smoking habits were more likely to suffer from severe COVID-19. Critically ill patients had lower lymphocyte counts and red blood cell counts, and higher white blood cell counts (P<0.05). Expectedly, serum inflammatory factors (NLR, PLR, LMR, CLR, PCT, CRP), coagulation markers (APTT, PT, TT, FIB, D-Dimer), Myocardial damage markers (hs-TNT, LDH) were significantly increased (P<0.05) in severe COVID-19 patients. Surprisedly, those patients showed obviously elevated levels of common tumor markers (ProGRP, CYFRA21-1, SCC, NSE) (P<0.05). In this case, the levels of tumor marker reflected more the condition of inflammation than the growth of tumor. More importantly, HA and PIIIN-P were highly associated with COVID-19 severity. The AUC of the ROC curve for the diagnosis of severe COVID-19 by HA and PIIIN-P was 0.826. Meanwhile, HA was positively correlated with myocardial damage markers (hs-TNT, LDH). PIIIN-P was positively correlated with myocardial damage markers (hs-TNT, LDH) and inflammatory factors (NLR, PLR, LMR, CLR, ProGRP, SCC, PCT, CRP). On the contrary, PIIIN-P was negatively correlated with pulmonary function indexes (oxygenation index and oxygen saturation of hemoglobin).

**Conclusion:**

HA and PIIIN-P are highly associated with disease severity and progression of COVID-19 and can be used as new markers for the prediction of severe COVID-19.

## 1 Introduction

2019 novel coronavirus pneumonia (COVID-19) is an acute infectious disease caused by a novel coronavirus strain classified as severe acute respiratory syndrome coronavirus 2 (SARS-CoV-2) (Andersen et al., 2020; Wu et al., 2022). The disease is highly infectious and seriously threatens human life (Qin et al,. 2021; Allan et al., 2022). Although most patients infected with SARS-CoV-2 usually show mild to moderate symptoms with spontaneous resolution and a good prognosis, a small number of patients with COVID-19 will develop severe pneumonia, pulmonary edema, acute respiratory distress syndrome or multi-organ failure, leading to intensive care unit (ICU) admission and high mortality (Guan and Zhong, 2020). Therefore, early identification of severe and critical patients can lead to early active treatment and reduce mortality. Therefore, we need to actively search for early clinical serum markers, effective treatment regimens and methods to prevent infection. Effective biomarkers will be helpful for screening, clinical management and prevention of serious complications, so as to achieve early detection, early treatment and early recovery.

Currently, numerous reports show that severe COVID-19 patients have different degrees of pulmonary fibrosis after recovery, which has a serious impact on the prognosis (Bharat et al., 2020; King et al., 2022). This may be related to collagen metabolites procollagen type III amino-terminal propeptide (PIIIN-P), hyaluronic acid (HA) (Su et al., 2017; Hellman et al., 2020). HA and PIIIN-P are the main components of extracellular matrix and have immunomodulatory effects. The deposition and degradation of HA and PIIIN-P are closely related to the activity of immune cells. PIIIN-P is dissociated from type III procollagen during the biosynthesis of type III collagen and is characteristic of the early stages of repair and inflammation (Bjermer et al., 1989). There was a statistically significant correlation between collagen turnover biomarkers and inflammatory biomarkers. Despite their modest association, it supports positive feedback between inflammation and fibrosis (Su et al., 2017). HA, produced by fibroblasts and connective-tissue cells, is a mucopolysaccharide that forms the extracellular matrix and is a major component of the alveolar extracellular matrix that promotes lung interstitial development (Morales-Nebreda et al., 2015; Esposito et al., 2017). In response to various viral and inflammatory stimuli, the increase of hyaluronic acid reflects the process of lung fibrosis. Some studies have found that HA and PIIIN-P are related to lung injury (Hallgren et al., 1985).

In this study, the levels of HA and PIIIN-P were evidently higher in severe COVID-19 patients, and were significantly correlated with myocardial damage, inflammation and pulmonary insufficiency. HA and PIIIN-P can be used as potential markers for predicting severe COVID-19 to help early identification and timely intervention and treatment.

## 2 Materials and methods

### 2.1 Patients

A total of 420 patients diagnosed with COVID-19 admitted to Kunshan Hospital of Traditional Chinese Medicine from November 2022 to January 2023 were included in this study. All patients were confirmed by at least one SARS-CoV-2 PCR-positive nasopharyngeal swab. Patients with previous or current history of cancer were excluded. For each patient, the demographic and baseline characteristics were obtained: age, sex, comorbidities, surgery history, smoking history, SARS-CoV-2 PCR results, date of symptom onset, hospitalization and discharge dates, intubation and extubation dates, and deceased date. According to the clinical classification criteria in the Diagnosis and Treatment of novel coronavirus pneumonia (trial version 10) (Lin and Li, 2020), COVID-19 patients are classified into four categories: 1) Mild, mild symptoms and no pneumonia manifestation; 2) Typical, fever, or respiratory symptoms and imaging manifestation of pneumonia; 3) Severe, having any of the three conditions: respiratory distress, respiratory rate ≥30 beats/min; means oxygen saturation ≤93% in a resting state; arterial blood oxygen partial pressure/oxygen concentration ≤300 mm Hg (1 mm Hg = 0.133 kPa); 4) Critical, having one of the three conditions: shock incidence; respiratory failure and requiring mechanical ventilation; admission to ICU with other organ function failure. All patients were classified into either the mild or severe group, the mild group contained mild and typical patients, while the severe group included severe and critically severe patients. On admission, all patients were classified as mild. In this study, 243 patients were classified as mild group and 177 patients were classified as severe group. All the patients were treated following the guidelines issued by the China National Health Commission (trial version 3-5) (Lin and Li, 2020). This study was approved by the Medical Ethics Committee of Kunshan Traditional Chinese Medicine Hospital. All data used in the study was anonymous, so the requirement for informed consent was waived.

### 2.2 Data collection

Laboratory analyses were conducted upon hospital admission for COVID-19 symptoms, before any treatment. Complete blood cell counts, and percentages included white cell count, red cell count, neutrophil count, lymphocyte count, monocyte count, platelet count, NLR (neutrophil/lymphocyte ratio), PLR (platelet/lymphocyte ratio), LMR (lymphocyte/monocyte ratio), CLR (C-reactive protein/lymphocyte ratio), CRP (C-reactive protein) were conducted by a hematology analyzer (Sysmex, Japan). Coagulation markers including APTT (activated partial thromboplastin time), PT (Prothrombin time), FIB (functional fibrinogen), TT (thrombin time), D-Dimer were analyzed by a coagulation analyzer (Sysmex, Japan). Myocardial damage markers including hs-TNT (high-sensitivity troponin T), LDH (lactate dehydrogenase) and CK-MB (creatine kinase-myocardial band), common tumor markers including ProGRP (progastrin-releasing peptide), CYFRA21-1(cytokeratin 19 fragment), SCC (squamous cell carcinoma), Ferritin and NSE (neuron-specific enolase), and inflammation marker PCT (Procalcitonin) were measured by a chemiluminescence immunoassay analyzer (Roche, USA). Fibrosis markers including HA (Hyaluronic acid), PIIIN-P (Procollagen III, N-terminal propeptide), IV-C (type IV collagen IV), and LN (laminin) were tested by a chemiluminescence immunoassay analyzer (Autobio, China).

### 2.3 Statistical analysis

Statistical analysis was performed using SPSS 26.0 (SPSS, Chicago, IL, USA) statistical software and GraphPad Prism 8.0 (GraphPad Software, San Diego, CA, USA). Kolmogorov–Smirnov test was conducted to test the normality of distribution. The non-normally distributed variables were evaluated by nonparametric test. The differences among groups were assessed using the chi-squared test for categorical variables. Spearman rank correlation coefficient was used for linear correlation analysis between groups. Univariate logistic regression analysis was used to evaluate the prognostic value of different biomarkers with the severity of COVID-19 as the dependent variable. To estimate the diagnostic value of the biomarkers, area under the Receiving operating characteristic (ROC) curve analysis was performed. Data were shown as median ± interquartile range. *P<0.05, ** *P*<0.01, *** *P <*0.001. *P*<0.05 was considered statistically significant.

## 3 Results

### 3.1 General clinical data in mild and severe COVID-19 patients

A total of 420 patients with COVID-19 were included in this study. The demographics and baseline characteristics of these patients on admission were shown in **Table 1**. Compared with the female patients, the male patients were more likely to suffer from severe COVID-19 (OR=2.31, 95%CI, 1.42-3.19, P <0.001). Besides, age (≥46 years) (OR=5.28, 95%CI, 2.18-12.8, P <0.001), diabetes (OR=2.080, 95%CI, 1.33-3.26, P = 0.001), smoking (OR=2.43, 95%CI, 1.21 – 4.86, P = 0.01) and hypertension (OR=1.87, 95%CI, 1.26-2.77, P = 0.02) were also risk factors of severe COVID-19. There is no significant correlation between alcohol drinking (OR=1.69, 95%CI, 069-4.18, P=0.25) and severe COVID-19. 

**Table 1:** Association of various risk factors (comorbidities) with mild and severe COVID-19 patients (Univariate analyses).

Risk factors	Mild (n, %)	Severe (n, %)	Univariate	*p*
			OR (95% CI)	
Gender				
Female	119 (49)	55 (31)	1(ref)	
Male	124 (51)	122 (69)	2.13 (1.42 - 3.19)	< 0.001
Age(years)				
≤45	38 (15.64)	6 (3.39)	1(ref)	< 0.001
≥46	205 (84.36)	171 (96.61)	5.28(2.18-12.80)	
Smoking				
No	226 (94)	153 (86.93)	1(ref)	
Yes	14 (6)	23 (13.07)	2.43 (1.21 - 4.86)	0.01
Drink				
No	223 (96.10)	161 (93.60)	1(ref)	
Yes	9 (3.90)	11 (6.40)	1.69 (0.69 - 4.18)	0.25
Hypertension				
No	135 (56.72)	73 (41.24)	1(ref)	
Yes	103 (43.28)	104 (58.76)	1.87 (1.26 - 2.77)	0.002
Diabetes				
No	188 (80.34)	116 (66.29)	1(ref)	
Yes	46 (19.66)	59 (33.71)	2.080 (1.33 - 3.26)	0.001

OR, odds ratio; CI, confidence interval; N, total number of patients; (n, %), the number; and percentage of patients with (Yes) or without (No) specified conditions.Missing data about Smoking (3 patients in mild group and 1 patient in severe group), Drink (11 patients in mild group and 5 patients in severe group), Hypertension (5 patients in mild group) and Diabetes (9 patients in mild group and 2 patients in severe group).

### 3.2 Laboratory characteristics in mild and severe COVID-19 patients

Differences of the laboratory findings including complete blood cell counts, coagulation markers, myocardial damage markers and common tumor markers between mild and severe COVID-19 patients were observed as the disease progressed. Compared with the mild patients, the severe patients showed higher neutrophil counts and lower lymphocyte counts, with statistically significant differences (P< 0.05). Moreover, red blood cell (RBC)-related indicators include RBC, hemoglobin (HB), hematocrit (HCT), mean corpuscular volume (MCV), mean corpuscular hemoglobin (MCH), and RBC distribution width (RDW) were notably decreased (P< 0.05) (shown in **Table 2**). These results are consistent with the large amount of reported data. It has long been known that lymphopenia may affect the host adaptive immune responses and impact the clinical course of acute viral infections and then related to the severity of COVID-19. Besides, anemia most frequently accompanies infection in varying degrees.

**Table 2:** Levels of blood cell Biomarker in mild and severe COVID-19 patients (Univariate analyses).

Clinical markers	Mild Median (25%-75%)	Severe Median (25%-75%)	Univariate	*p*	AUROC
			OR (95% CI)		(95% CI)
White blood cell markers					
WBC (×10^9^/L)	5.9 (4.5, 8.2)	7.8 (5.7, 10.8)	1.16 (1.91 - 1.23)	< 0.001	0.65(0.59 - 0.70)
Neutrophil(×10^9^/L)	3.94 (2.86, 6.08)	6.48 (4.23, 9.29)	1.24 (1.16 - 1.33)	< 0.001	0.70 (0.65 - 0.75)
lymphocyte(×10^9^/L)	1.11 (0.81, 1.58)	0.67 (0.45, 0.97)	0.23 (0.15- 0.36)	< 0.001	0.75(0.70 - 0.80)
Monocyte(×10^9^/L)	0.5 (0.36, 0.70)	0.45 (0.29, 0.68)	0.91 (0.50 - 1.61)	0.76	0.45(0.39 - 0.50)
Red blood cell markers					
RBC(×10^9^/L)	4.14 (3.74, 4.49)	3.92 (3.48, 4.33)	0.59 (0.43 - 0.80)	< 0.001	0.41 (0.35 - 0.47)
HB (mg/ml)	124 (113, 134.5)	120 (106, 133)	0.99 (0.98 - 0.997)	0.01	0.43 (0.38 - 0.49)
HCT (%)	0.38 (0.34, 0.41)	0.36 (0.32, 0.4)	0.01 (0.00 - 0.50)	0.02	0.43 (0.38 - 0.49)
MCV (fL)	91.2 (88.2, 93.75)	92 (88.7, 95.7)	1.06 (1.02 - 1.10)	0.00	0.57 (051 - 0.62)
MCH (pg)	30.2 (29.2, 31.25)	30.5 (29.2, 31.7)	1.10 (1.01 - 1.20)	0.03	0.55(0.49 - 0.60)
RDW (%)	12.5 (12, 13.1)	13.1 (12.5, 13.9)	1.34 (1.16 - 1.55)	< 0.001	0.67 (0.62 - 0.73)
MCHC (mg/ml)	332 (325, 339)	331 (321, 342)	0.99 (0.98 - 1.01)	0.56	0.49(0.43 - 0.55)
Platelet markers					
PLT (×10^9^/L)	213 (156, 271.5)	198 (143, 253)	0.99 (0.996 - 1.00)	0.08	0.44(0.39 - 0.50)
MPV (fL)	10.9 (10.2, 11.7)	11 (10.3, 11.8)	1.12 (0.93 - 1.34)	0.24	0.53(0.48 - 0.59)

WBC, white blood cell; RBC, erythrocytes; HB, hemoglobin; HCT, hematocrit; MCV, mean corpuscular volume; MCH, mean cell hemoglobin; MCHC, mean cell hemoglobin concentration; RDW, red blood cell distribution width; PLT, platelets; MPV, mean platelet volume.

There is consensus that severe COVID-19 infection activates systemic inflammatory response, and the degree of inflammation is significantly correlated with the prognosis of the disease. NLR, PLR, LMR, CLR, PCT and CRP were commonly used to evaluate the systemic inflammation. Compared with mild COVID-19 patients, severe patients showed raised values of NLR (OR 1.21, P <0.001), PLR (OR 1.01, P <0.001), LMR (OR 0.63, P <0.001), CLR (OR 1.01, P <0.001), PCT (OR 1.09, P =0.01) and CRP (OR 1.02, P <0.001) (**Table 3**).

**Table 3:** Levels of Inflammatory Biomarker in mild and severe COVID-19 patients (Univariate analyses).

Inflammation markers	Mild Median (25%-75%)	Severe Median (25%-75%)	Univariate	*p*	AUROC
			OR (95% CI)		(95% CI)
NLR	3.65 (2.29, 6.05)	8.79 (5.11, 17.47)	1.21 (1.15 - 1.27)	< 0.001	0.79 (0.75 - 0.84)
PLR	176.34 (135.48, 248)	282.14 (177.5, 418.84)	1.01 (1.00 - 1.01)	< 0.001	0.71 (0.69 - 0.76)
LMR	2.31 (1.64, 3.30)	1.51 (0.98, 2.34)	0.63 (0.52 - 0.75)	< 0.001	0.69 (0.64 - 0.74)
CLR	15.78 (4.39, 40.91)	66.61 (22.71, 160.25)	1.01 (1.01 - 1.02)	< 0.001	0.76 (0.71 - 0.81)
PCT (ng/mL)	0.04 (0.02, 0.09)	0.195 (0.06, 0.65)	1.09 (1.02 - 1.16)	0.01	0.78 (0.74 - 0.83)
CRP (mg/L)	14.5 (4.45, 37.55)	42.8 (16.9, 103.75)	1.02 (1.01- 1.02)	< 0.001	0.72 (0.67 - 0.77)

NLR, Neutrophils to Lymphocytes Ratio; PLR, platelet to lymphocyte ratio; LMR, Lymphocytes to Monocytes Ratio; CLR, C-reactive protein to Lymphocytes Ratio; PCT, procalcitonin; CRP, C-reactive protein; IL-6, interleukin 6.

Acute inflammation, as a response to severe infection, results in a systemic activation of the coagulation system. In this study, the values of APTT, PT, TT, FIB and D-Dimer were significantly higher in severe COVID-19 patients, compared with those mild patients (P <0.05) (**Table 4**). Hypertension, arrhythmia, cardiomyopathy and coronary heart disease are amongst major cardiovascular disease comorbidities seen in severe cases of COVID-19. Myocardial injury is one of the important pathogenic features of COVID-19 (Rusu et al., 2022). Consistent with this phenomenon, severe COVID-19 patients in our study showed high levels of hs-TNT (OR 1.07, P<0.001), LDH (OR 1.01, P<0.001) but not CK-MB. To our surprise, some traditional tumor markers, such as serum CYFRA21-1(OR 1.53, P <0.001), ProGRP (OR 1.08, P =0.01), SCC (OR 1.54, P =0.01) and NSE (OR 1.07, P =0.02) were significantly increased in severe patients (**Table 4**). In this case, the levels of tumor marker reflected more the condition of inflammation than the growth of tumor.

**Table 4:** Serum values of various laboratory/clinical markers in mild and severe COVID-19 patients (Univariate analyses).

Clinical markers	Mild	Severe	Univariate	*p*	AUROC
			OR (95% CI)		(95% CI)
Coagulation markers					
APTT (s)	28.2 (26.1, 31.8)	30.25 (26.7, 34.9)	1.08 (1.04 - 1.13)	< 0.001	0.60 (0.54- 0.65)
PT (s)	12.9 (12.5, 13.6)	13.45 (12.7, 14.4)	1.29 (1.12 - 1.50)	< 0.001	0.62 (0.57 - 0.68)
FIB (g/L)	4.72 (3.85, 5.69)	5.1 (3.93, 6.27)	1.15 (1.02 - 1.30)	0.03	0.56 (0.50 - 0.61)
TT (s)	16.9 (16.1, 17.6)	17.3 (16.48, 18.33)	1.34 (1.17 - 1.54)	< 0.001	0.61 (0.55 - 0.66)
D-Dimer (mg/L)	0.55 (0.31, 0.98)	1.52 (0.66, 3.73)	1.38 (1.21 - 1.56)	< 0.001	0.75 (0.70 - 0.80)
Myocardial damage markers					
hs-TNT (pg/mL)	7.5 (4.85, 13.28)	17.3 (9.88, 34.55)	1.07 (1.04 - 1.11)	< 0.001	0.76 (0.69 - 0.83)
LDH (U/L)	236.9 (200.05, 293)	317 (254, 404.1)	1.01(1.01 - 1.01)	< 0.001	0.74 (0.70 - 0.79)
CK-MB (U/L)	7.0 (3, 10.25)	9.0 (5, 14)	1.02 (0.99 - 1.05)	0.27	0.60 (0.50 - 0.70)
Tumor markers					
ProGRP (pg/mL)	25.4 (17.05, 30)	42.4 (29.74, 67.70)	1.08 (1.02- 1.14)	0.01	0.78 (0.64 - 0.92)
CYFRA21-1 (ng/mL)	2.57 (1.89, 4.15)	6.11 (4.19, 8.84)	1.53 (1.26 - 1.86)	< 0.001	0.83(0.75 - 0.90)
SCC (ng/mL)	0.9 (0.6, 1.4)	1.5 (1.0, 2.88)	1.54 (1.13 - 2.11)	0.01	0.72 (0.63 - 0.81)
Ferritin (μg/L)	595.65(370.85,820)	807.57(445-935.5)	1.001(1.00 -1.002)	0.05	0.59 (0.49 - 0.68)
NSE (ng/mL)	14.59(10.75,16.65)	21.45(12.2,21.25)	1.07(1.01 -1.14)	0.02	0.63 (0.54 - 0.73)

APTT, activated partial thromboplastin time; PT, Prothrombin time; FIB, functional fibrinogen; TT, thrombin time; hs-TNT, high-sensitivity troponin T; LDH, lactate dehydrogenase; CK-MB, creatine kinase-myocardial band; ProGRP, progastrin-releasing peptide; CYFRA21-1, cytokeratin 19 fragment; SCC, squamous cell carcinoma; NSE, neuron-specific enolase.

**Figure 1:**
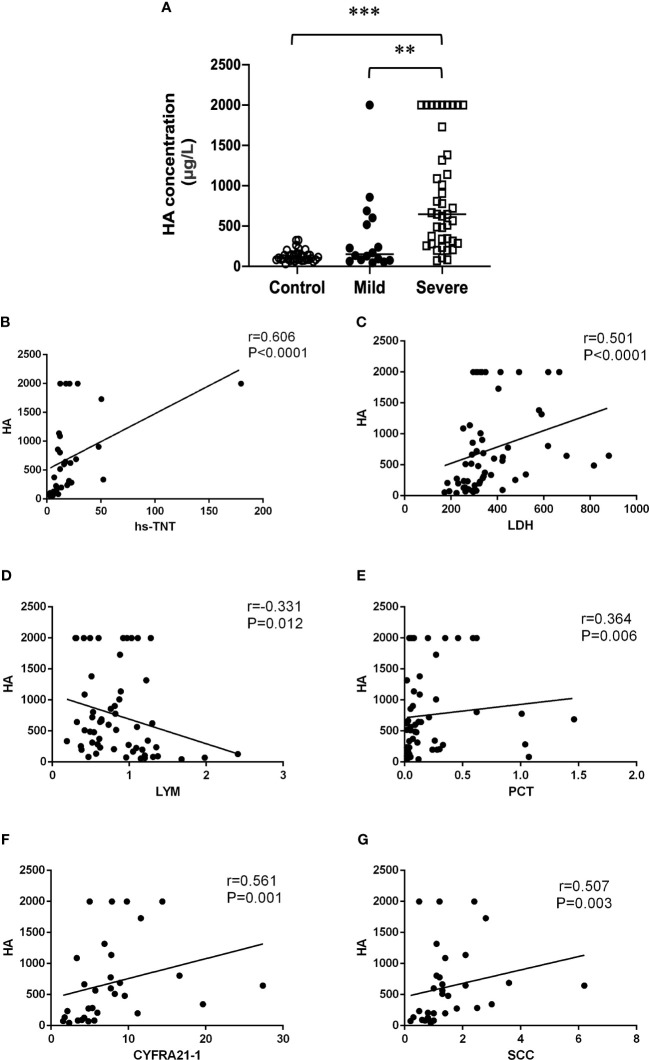
**(A)** Dot plot of serum concentration of HA in control, mild and severe COVID-19 patients. Correlations between HA and hs-TNT **(B)**, LDH **(C)**, Lymphocytes **(D)**, PCT **(E)**, CYFRA21-1 **(F)**, SCC **(G)**. ** *P*<0.01, *** *P*<0.001.

**Figure 2:**
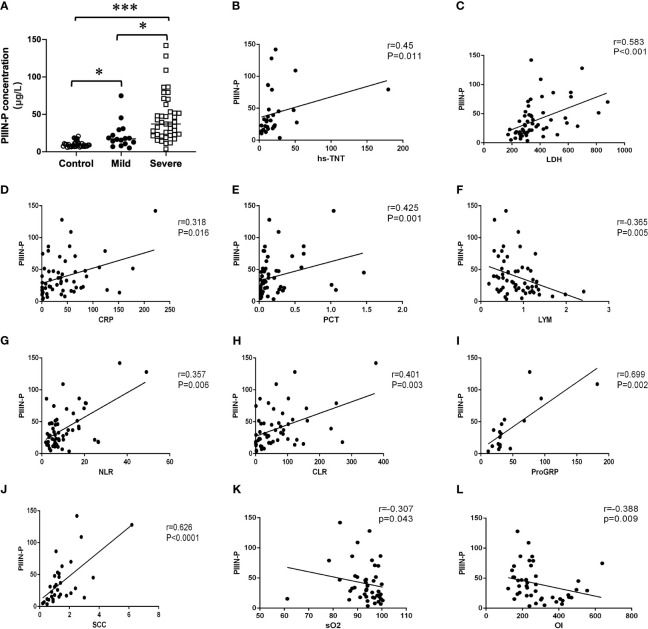
**(A)** Dot plot of serum concentration of PIIIN-P in control, mild and severe COVID-19 patients. Correlation between PIIIN-P and hsTNT **(B)**, LDH **(C)**, CRP **(D)**, PCT **(E)**, Lymphocytes **(F)**, NLR **(G)**, CLR **(H)**, ProGRP **(I)**, SCC **(J)**, sO2 **(K)**, OI **(L)**. OI, oxygenation index; sO2, oxygen saturation of hemoglobin. * *P*<0.05, *** *P*<0.001.

Interestingly, we found that HA and PIIIN-P, which contributed to fibrosis-related processes, in severe patients were significantly higher than those in mild patient (P<0.05) (**Figures 1A**,**2A**). Furtherly, the correlations of HA and PIIIN-P with myocardial injury markers and inflammation markers in COVID-19 patients were analyzed. The results showed that HA was and positively correlated with hs-TNT (r = 0.606, P <0.001), LDH (r = 0.501, P <0.001), CYFRA21-1 (r = 0.561, P = 0.001), SCC (r = 0.507, P = 0.003), and negatively correlated with lymphocytes (r = -0.331, P = 0.012) (**Figures 1B-G**). Similarly, PIIIN-P was positively correlated with ProGRP (r = 0.699, P = 0.002), SCC (r = 0.626, P<0.001), LDH (r = 0.583, P <0.001), hs-TNT (r = 0.45, P = 0.011), and negatively correlated with SO_2_ (r = -0.307, P = 0.043), OI (r = -0.338, P = 0.009) and lymphocytes (r =-0.365, P = 0.005) (**Figures 2B-L**). These results indicated that HA and PIIIN-P were certainly related to clinical symptoms, and may play an important role in the prognosis of COVID-19.

### 3.3 ROC curve of HA and PIIIN-P for predicting severe COVID-19 patients

**Figure 3:**
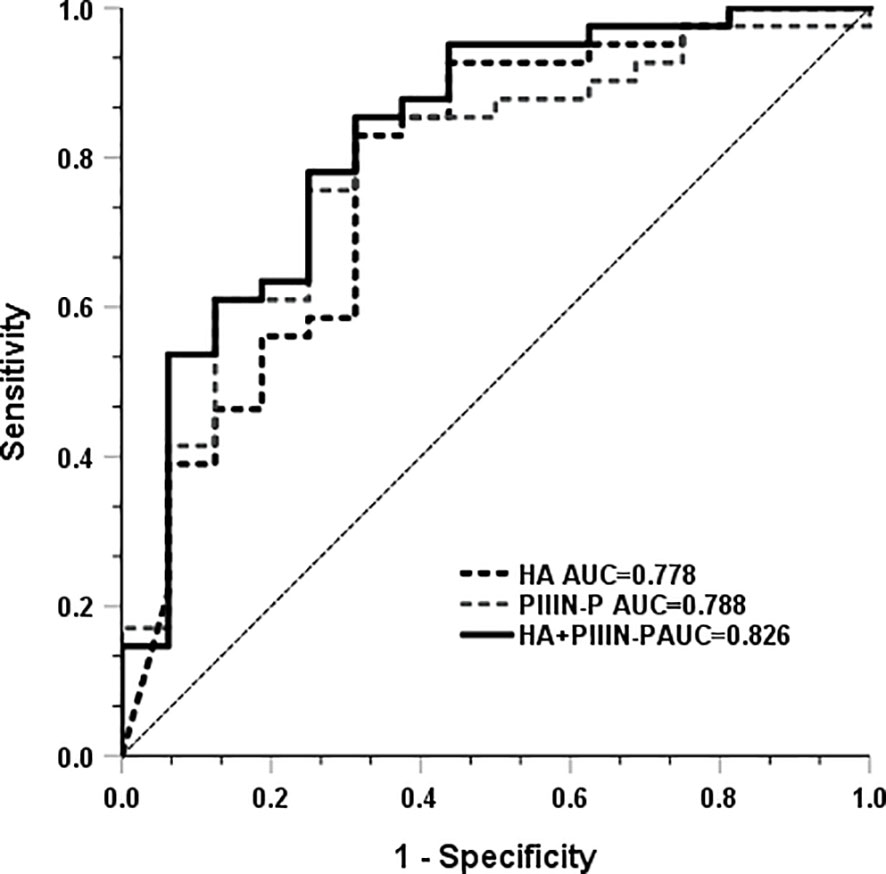
Areas under the curves of HA, PIIIN-P and HA + PIIIN-P.

Subsequently, we analyzed whether HA and PIIIN-P could be used as predictors of COVID-19 disease progression. The results showed that the AUC of HA and PIIIN-P in predicting the risk of severe disease in patients with COVID-19 was 0.778 and 0.788, respectively. The AUC of the combined of HA and PIIIN-P to predicting severe COVID-19 was 0.826, accompanied by 85.4% sensitivity and 68.8% specificity. The Youden index was 0.737, and the 95% confidence interval was 0.795-1, as shown in **Figure 3**. The results suggested that the combination of HA and PIIIN-P could be used as a predictor of COVID-19 disease progression.

## 4 Discussion

High inflammation in COVID-19 disease is known to be a major cause of poor patient outcomes. In this study, we retrospectively summarized a series of clinical laboratory tests in the serum of patients with mild and severe COVID-19. The results showed that the levels of WBC, neutrophil, PCT, CRP, NLR, PLR, CLR, D-dimer, LDH and hs-TNT, SCC were significantly higher in severe COVID-19 patients than those in mild COVID-19 patients. However, lymphocytes, erythrocytes, and FIB were significantly decreased, which was consistent with previous reports. However, few reports have reported that HA and PIIIN-P can also be used as serum markers for the prognosis of COVID-19. Our data showed that HA and PIIIN-P in severe COVID-19 patients were significantly higher than those in mild patients. Simultaneously, HA, PIIIN-P and SCC, hs-TNT, LDH, PCT, CRP, NLR and other serum markers were positively correlated, and these serum markers were the key factors related to the severity of the disease. These results suggest that HA and PIIIN-P may regulate a variety of biological and pathological processes involving inflammatory response, immune response and tissue damage during the occurrence and development of COVID-19. More importantly, the combination of HA and PIIIN-P had a high area under the curve in the diagnosis of severe COVID-19 patients. Our findings provide insights into the detailed pathological process of COVID-19 in patients. This will not only promote understanding about the molecular pathology of the disease and facilitate early diagnosis, but also assist in the evaluation of patients’ prognosis.

As consistent with previous reports, most of the COVID-19 patients are middle-aged and elderly men. In this study, 96.61% of the severe COVID-19 patients are ≥46 years old. Male patients who have smoking habits are more likely to suffer from severe COVID-19. As reported, smoking can increase the expression of angiotensin-converting enzyme 2 (ACE2) in lung tissues, which is one of the binding receptors of SARS-CoV-2 (Ponti et al., 2020). Additionally, old age, which is accountable for reduced effectiveness in the immune system, is partially responsible for the increased prevalence and severity of infectious diseases (Gunn et al., 2022). This could explain why the male, elderly patients and those with any comorbidities, especially with hypertension and diabetes, were more likely to develop into severe cases.

Lymphocytes play an important role in the body’s anti-virus process by enhancing immunity (Diao et al., 2020). Our data depicted that white blood cell counts, and neutrophil counts were significantly increased in most patients with severe COVID-19. On the contrary, the lymphocyte count of severe patients was lower than that of mild patients. Furthermore, NLR, PLR, LMR and CLR not only effectively reflect the dynamic relationship between inflammation and immune regulation *in vivo*, but also have a higher predictive value for the short-term poor prognosis of COVID-19 patients (Guthrie et al., 2013; Seyit et al., 2021). In this study, NLR, PLR, LMR, CLR in severe COVID-19 patients were perceptibly higher than those in mild COVID-19 patients, indicating that NLR, PLR, LMR and CLR were risk factors for severe COVID-19. In addition, common inflammatory markers such as CRP and PCT were also increased in most patients, especially severe patients, indicating that there was a serious inflammatory response in the severe patients. It is reasonable that SARS-CoV-2 infection induces excessive release of inflammatory factors, aggravates lung tissue damage, and leads to respiratory failure and death (Hosseninia et al., 2023). Therefore, elevated values of NLR, PLR, LMR, CLR, CRP and PCT in severe patients may be associated with poor prognosis.

Disseminated intravascular coagulation (DIC) was found in most severe and fatal patients with SARS-CoV2 infection (Wichmann et al., 2020). It has been reported that in patients with severe COVID-19, D-dimer and FIB are especially increased, indicating that these patients have coagulation activation and secondary hyperfibrinolysis (Levi et al., 2020). In this study, the prolongations of APTT, PT and TT were frequently observed in severe COVID-19 patients. The elevated levels of FIB and D-Dimer were associated with the prognosis of COVID-19. The findings are largely consistent with previous reports. Some studies have shown that systemic inflammatory response syndrome and microvascular thrombosis triggered by cytokine release syndrome in COVID-19 patients may be important mechanisms for the development of COVID-19 related to myocardial injury (Du et al., 2020; Ponti et al., 2020). Unsurprisingly, hs-TNT and LDH were conspicuously higher in severe patients than in mild patients in this study.

In most cases, tumor markers are employed to predict primary or secondary tumor risk. For example, ProGRP and CYFRA21-1 are common biomarkers of small cell lung cancer, while SCC is a type of tumor-associated antigen related to squamous cell lung cancer (Hirakata et al., 1995). In this study, ProGRP, CYFRA21-1 and SCC in severe COVID-19 patients were significantly higher than those in mild patients. Given patients with previous or current history of cancer were excluded in this study, ProGRP, CYFRA21-1, SCC reflected more the condition of inflammation than the tumor risk. It is believed that inflammatory responses from benign conditions can cause non-cancer-related elevations in tumor markers (Gunn et al., 2022).

Acute respiratory distress syndrome (ARDS) is one of the most prominent clinical symptoms of severe COVID-19 patients (Jiang et al., 2020). The proliferation and activation of fibroblasts after lung injury cause the increased secretion of HA, which accumulates a large amount of HA in the lungs of ARDS patients (Halllgren et al., 1989; Xu et al., 2020). HA is an important component of extracellular matrix, which plays a unique signal role in tissue development, remodeling and repair (Petrey and de la Motte, 2014). Simultaneously, HA regulates a variety of biological and pathological processes involving inflammation, immune response and tissue damage (Liang et al., 2016). Increasing studies suggest that HA may be associated with the clinical symptoms of COVID-19 (Ding et al., 2020; Hellman et al., 2020), which is consistent with our study. HA was found to be significantly higher in patients with severe COVID-19 than in patients with mild COVID-19. Notably, our results showed that HA was positively correlated with myocardial injury markers such as hs-TNT and LDH, and inflammatory markers such as CYFRA21-1, SCC. These findings suggest that HA was associated with the progression of COVID-19 and further support that HA can be a novel biomarker for predicting the clinical progression of COVID-19.

There are few report about the relationship between PIIIN-P and COVID-19. PIIIN-P is a precursor of type III collagen, which is a good indicator of connective tissue metabolism. PIIIN-P is also the main extracellular matrix protein of the lung, which is distributed in the lung interstitium, bronchus and blood vessels (Lennon and Singleton, 2011). It was reported that PIIIN-P was increased in the early stage and active stage of pulmonary fibrosis, so the determination of PIIIN-P can be used as a good index for early detection of pulmonary fibrosis (Low et al., 1992). It has been reported that excessive deposition of collagen in patients with COVID-19, especially in severe patients, can lead to pulmonary fibrosis, leading to dysfunction of lung ventilation and gas exchange, causing respiratory failure and irreversible damage (Tran et al., 2022; Patrucco et al., 2023). The results of this study communicated that PIIIN-P in severe patients was significantly higher than that in mild patients. Furthermore, PIIIN-P was positively correlated with CRP, PCT, CLR, NLR, SCC and ProGRP suggesting that the increase of inflammation may accompany and promote the progress of pulmonary fibrosis. Therefore, lung function index including SO2 and OI was negatively correlated with the level of PIIIN-P. The above results further establish that PIIIN-P can better reflect the severity of COVID-19, especially the respiratory complications.

In addition to respiratory system damage, cardiac complications such as myocardial injury and heart failure are also common in patients with COVID-19 infection. The auxiliary examination shows abnormal troponin or myocardial enzyme spectrum (Azevedo et al., 2021). PIIIN-P is an important component of cardiomyocytes and myocardial interstitium, which plays a crucial role in the maintenance of cardiac structure, force transmission between myocardium and the maintenance of myocardial contractility (Dupuy et al., 2019). Studies have found that PIIIN-P is an important risk factor for cardiac function and can better reflect the degree of myocardial fibrosis and heart failure, and its expression is increased in acute myocardial infarction (Wellens, 2019). Coincidentally, PIIIN-P was positively correlated with hs-TNT and LDH. Although the association between them was not significant, it could help to support the important role of PIIIN-P in the process of cardiovascular injury. These results suggest that PIIINP plays a key role in the progression of COVID-19, and can be used as a marker for the progression of COVID-19, and its dynamic changes may be used to detect the progression of COVID-19.

In conclusion, our study provides new insights into the potential clinical application of HA and PIIIN-P as suitable markers for COVID-19 patients. It reveals that changes in serum HA and PIIIN-P were significantly associated with clinical outcomes.

## Data availability statement

The original contributions presented in the study are included in the article/supplementary materials, further inquiries can be directed to the corresponding author/s.

## Ethics statement

This study was approved by the Medical Ethics Committee of Kunshan Traditional Chinese Medicine Hospital. All data used in the study was anonymous, so the requirement for informed consent was waived.

## Author contributions

CH, and TY designed the study. TY, LL carried out the experiments. XW and JX provide technical support. CH and TY wrote and read the manuscript, and all authors reviewed the manuscript. All authors contributed to the article and approved the submitted version.
